# Improving psychotherapies offered in public hospitals in Nairobi, Kenya: extending practice-based research model for LMICs

**DOI:** 10.1186/s13033-018-0254-7

**Published:** 2018-12-11

**Authors:** Manasi Kumar, Mary Wangari Kuria, Caleb Joseph Othieno, Fredrik Falkenström

**Affiliations:** 1 Department of Psychiatry, College of Health Sciences, University of Nairobi, P.O. Box 19676, Nairobi, 00202 Kenya; 2 Research Department of Clinical Health and Educational Psychology, University College London, Gower Street, London, WC1E 6BT UK; 3 Department of Behavioural Sciences and Learning, Linköping University, Linköping, Sweden

**Keywords:** Psychotherapies, Mental illness, Practice-informed research, Implementation challenges, Kenya, Poverty

## Abstract

**Background:**

Psychotherapy and mental health services in Nairobi’s public hospitals are increasing. Rather than prematurely imposing psychotherapy protocols developed in Western countries to Kenya, we argue that first studying psychological interventions as they are practiced may generate understanding of which psychological problems are common, what interventions therapists use, and what seems to be effective in reducing psychiatric problems in a lower and middle income country like Kenya.

**Method:**

We present preliminary findings from a process-outcome study involving 345 patients from two public institutions, Kenyatta National and Mathare National Hospitals. We asked our patients to fill out a brief personal information questionnaire, Clinical Outcomes in Routine Evaluation-Outcome Measure (Evans et al. in Br J Psychiatry 180:51–60, 2002, and the Session Alliance Inventory (Falkenström et al. in Psychol Assess 27:169–183, 2015) after each session. We present descriptives for CORE-OM, patient-therapist concordance on the SAI, and using longitudinal mixed-effects model, test change in CORE-OM over time with various therapy and patient factors as predictors in regression analyses.

**Results:**

The majority of patients who attended the outpatient care clinics were young males. Our regression analysis suggested that patients with depression reported higher initial distress levels (2.75 CORE-OM scores, se = 1.11, z = 2.48, *p* = 0.013, 95% CI 0.57–4.93) than patients with addictions, anxiety, or psychosis. Older clients improved slower (0.08 CORE-OM scores slower improvement per session per year older age; se = 0.03, z = 3.02 *p* = 0.003, 95% CI 0.03, 0.14). Female patients reported higher initial distress than men (2.62 CORE-OM scores, se = 1.00, z = 2.61, *p* = 0.009, 95% CI 0.65, 4.58). However, interns had patients who reported significantly higher initial distress (3.24 CORE-OM points, se = 0.90, z = 3.60, *p* < 0.001, 95% CI 1.48, 5.00), and improved more over time (− 1.20 CORE-OM scores per session, se = 0.51, z = − 2.35, *p* = 0.019, 95% CI − 2.20, − 0.20) than patients seeing mental health practitioners. The results showed that at average alliance, CORE-OM decreased by 1.74 points per session (se = 0.21, *p* < 0.001). For each point higher on the SAI at session 2, the CORE-OM decreased by an additional 0.58 points per session (se = 0.25, *p* = 0.02).

**Discussion:**

Our objective was to study psychotherapies as they are practiced in naturalistic settings. The overall significant finding is that our participants report improvement in their functioning mental health condition and distress reduced as psychotherapy progressed. There were many more male than female participants in our sample; younger patients improved more than older ones; and while interns had patients with higher distress, their patients improved better than those patients attended by professionals.

**Conclusions:**

These are preliminary observations to consider for a larger sample follow-up study. Before changing practices, evaluating the existing practices by mapping clinical outcomes is a helpful route.

## Introduction

### Understanding psychotherapy in a low-resource health services context

Mental health problems form a significant burden of disease worldwide [43]. In developing countries, the management of psychosocial problems is hampered by inadequate facilities and under qualified staff. Several strategies have been put in place to counter this: (a) training more professionals and increasing the workforce, such as clinical psychologists, psychiatric social workers, and psychiatrists in peri-urban and rural areas [27]; (b) task-sharing or task-shifting [5, 50] where increasingly, community health workers are providing community outreach, initial assessment, and screening, and in a few pioneering programs, also providing simple psychosocial care interventions; (c) utilizing an assortment of staff using approaches to provide psychiatric and psychosocial treatments instead of a monotherapy model [4, 45–48]. The evidence base for these approaches is being vigorously discussed and tested in global mental health platforms (see [6, 7, 11, 26]. Global mental health research shows that the eclectic approaches, when used in a systematic and moderated manner and when attending to systemic barriers and communication, can yield good results in treatment of common mental disorders (CMDs) [33, 37, 40]. An assortment of staff, who can task-shift and task-share, is more sustainable in low-resource settings than using specialists alone [35, 54]. In Kenya, as in other LMIC contexts, the strong mobilization of an eclectic tool-kit combining several evidence based techniques, that is known as a common elements treatment approach [39]. While the approach brings in the best strategies adding to the treatment enhancement, it also makes it harder to study the efficacy of one kind of intervention over another.

### Mental Health Services in Kenya

We have been working on addressing services and mental health treatment-related gaps in the University of Nairobi mental health clinics and two government-funded facilities, Kenyatta National Hospital (KNH) and Mathare Psychiatric Hospital (MPH) since 2013 (Falkenström et al. 2016). Our team of two senior psychiatrists and two clinical psychologists have been working on developing a conceptual framework to understand and appraise psychotherapy, mental health, and psychosocial treatments offered in these facilities. Additionally, two psychiatrists and a clinical psychologist from our team at the University of Nairobi have been working on mental health systems capacity building and identifying common treatment elements that can be used to strengthen mental health care in the two hospitals.

Several modes of psychotherapies and counseling are practiced in Kenya on a variety of clinical conditions, the most common being depression, phobia and panic disorders, generalized anxiety disorder, mixed anxiety and depression, adjustment disorder, dissociative disorder, unexplained somatic symptoms, sleep problems, and substance-use disorders [27–30]. Internationally, the efficacy of a number of psychotherapies has been established, especially for disorders such as PTSD, depression, and anxiety disorders [18, 42–44]. However, local conditions or cultural differences may affect the efficacy of psychotherapy in unpredictable ways and necessitate translational research. Although the effectiveness of psychotherapy in Kenya and sub-Saharan Africa has been demonstrated for some treatment modalities, such as cognitive behavioral therapy and brief interventions in the treatment of alcohol dependence [41], several gaps exist in the overall mental health services evidence base for most of the psychotherapies currently being used in East Africa, such as group interpersonal therapy [3, 40, 51], trauma-focused CBT for orphaned children [10], and family-based CBT for youth [31] for integration and scaling up in routine care in public hospitals. In addition, highly controlled studies do not take into account differential organizational structures and the particular approaches that may be used by therapists to deliver psychotherapy in hospital or community-based settings [22, 33]. Therefore, pragmatic trials and systems studies on diffusion of innovative findings have become pertinent. If care and health are to be improved, research must be designed, disseminated, and implemented in collaboration with stakeholders [22]. Psychotherapy, either alone or in combination with psychopharmacology has been shown to be effective in treating psychological disturbances [8]. Although the efficacy of psychotherapy is likely to be universal, its cultural adaptation, modification for local health, and community contexts in different geopolitical settings has been an ongoing work for several decades now [52, 54]. Blending efficacy, effectiveness, and implementation research has produced evidence with greater relevance to mental health practice and policy [1, 37, 40].

### Extending a naturalistic design to study effectiveness of existing psychotherapies

The field of psychotherapy research is divided around which research design is most suitable for the study of psychotherapy and mental health interventions. Currently the most influential research has taken the blueprint from medical research, using the randomized controlled trial (RCT) as the gold standard. Although strong for establishing causality, many researchers have noted that the RCT is often limited in external validity: i.e. in the generalization to the patients, therapists, and settings of usual care [13]. In the LMIC settings, implementation of treatments and whether these are evidence-based have now become areas of great debate [39].

An alternative research design that has gained momentum in psychotherapy research focuses on the process and outcome of treatments as they are conducted in routine clinical practice (e.g., [9, 19, 20, 24]. In the implementation science discourse this is termed as practice-based evidence. In practice-based evidence research, one strand of thinking is to effectively blend what works well on ground with what needs to be practice-wise altered. Another strand is that the process of evaluation further involves identification of potential and actual influences on the conduct and quality of implementation [2] of existing services. In addition to enhancing ecological and external validity, research findings may be easier to communicate to practitioners since data is more practice-orientated than findings from laboratory conditions or new designs that alter practice dramatically. The added challenge is that the interventions that are not locally relevant and culturally consonant may generate negative effects including inappropriate diagnoses and interventions, increased stigma, and poor health outcomes [32]. The dominant strategy for linking academic psychotherapy research to clinical practice so far has been through dissemination of RCT findings to practicing therapists. We have discussed the extreme form of “empirical imperialism” [34] where researchers impose their views on what practitioners should do regardless of the needs and opinions of practitioners and patients in another paper [15]. Disseminating information, changing practices, and influencing public policy on improved access to mental health care in LMIC are complex issues and each needs to be carried out alongside the other. For instance, it is likely that some therapists and clinics are already achieving good results with their patients, or at least with some groups of patients. It would then be unnecessary for them to change their practice on the basis of evidence established in other settings that has not been well-tested locally. Additionally, dissemination without regard to patient and clinician preferences is likely to be unsuccessful. The harmful effects of disseminating evidence-based therapies developed in Western cultures to non-Western countries without regard to local organizational and socio-cultural conditions or health system capacities is probably even higher, since local therapists are likely to have already modified their methods to cultural conditions [40].

## Aim and objectives

In this paper we aim to share the findings from the first phase of our study where we tap into the ongoing process and outcome of psychotherapies offered at two university hospitals. Our key objectives are to study the effectiveness of psychotherapy as currently being delivered to maximize clinical utility for clinicians and bolster mental health services delivery.

## Methods

Our study received approval from University of Nairobi and Kenyatta National Hospital ethical review board (approval no. KNH-ERC/A/162). This is an ongoing naturalistic, observational study aiming to collect data on the range of psychological interventions practiced and in this exploratory paper we present preliminary findings from 345 patients. Any intervention described by practitioners as ‘counselling’ or ‘psychotherapy’ was included. Patients were recruited after their first appointment. No rewards were given for participation. Therapists recorded participants’ demographic details and diagnosis. Our inclusion criteria was patients above 18 years of age, able to give consent, no history of mental retardation and be willing to fill out all therapy process forms. Therapists were included in the study if they agreed to fill out patient process and outcome forms, and signed written informed consent. The self-report questionnaire of CORE-OM [12] was completed by the patient, in English or Kiswahili, before each session. The Session Alliance Inventory [14], a six-item questionnaire asking about working alliance, was filled out by patients after every session like CORE-OM. Data was extracted from clinical notes by two psychiatrists, two clinical psychologists, and three research assistants. This is an ongoing work, but some pilot data shows emerging differences from published studies in UK, Europe, and Sweden, which are presented here for discussion.

## Integrating practice-based research to understand dissemination and implementation challenges

Our strategy starts with a practice-based approach: gathering data from routine clinical practice by studying what exists on the ground and looking at patterns that indicate success versus failure. Once enough data has been collected it is analyzed to identify subgroups of patients who are not improving at expected rates. After identifying subgroups of patients with inferior outcome, a third phase would ensue in which researchers search for the treatment(s) with the best research evidence for the identified patient group. Therapists will then be trained in this best-practice approach, and a clinical trial would be conducted to test if this treatment is superior to treatment as usual with these particular patient subgroups. This strategy has the advantage of first investigating the effectiveness of treatment as usual before disseminating evidence-based treatments, avoiding the risks inherent in ‘blind dissemination’. Another advantage is to build on capabilities, approaches, and competencies that the local culture has managed to cultivate to suit its complex and unique needs.

In this model, akin to dissemination and implementation science approaches and psychotherapy effectiveness models, we use both qualitative and quantitative methods to complement each other. Our qualitative work [36] is helping us understand Kenyan patients’ illness, cure perceptions, and attributions. Our ongoing work with therapists on identifying barriers to providing care that include structural, practical, professional, and personal challenges and difficulties would become basis to understanding the process and outcome data we have collected from therapists and patients in the quantitative work. The quantitative studies aim to capture the phenomena of where care is bolstered and where it fails in looking at both patients’ and therapists’ inputs at the level of psychopathology and time in therapy. We have built-in interfaces with clinic in-charges and therapists in our study such that these findings can be relayed back to them for inputs and feedback from the practitioners’ end. This approach is also time efficacious in that changes to practice are only implemented if data tells us they need to be changed [15].

## Setting and procedure

Data was collected at the Mathare National Hospital (MNH), a specialist psychiatric referral hospital, and the outpatient psychiatric clinic, mental health department and youth clinic of Kenyatta National Hospital (KNH), a 1500-bed national teaching and referral hospital. The latter two departments at KNH offer psychiatry, clinical psychology, social work, and counselling to adults and adolescents respectively. On average KNH sees 240 adult patients per month (about 60 patients per week) and MNH sees 320 adult patients in a month (about 80 per week) in various clinics and outpatient services offering mental health services.

Three research assistants working part-time in the project approached patients in the waiting room of the clinics where they described the study and asked patients if they were willing to participate in the study by filling out questionnaires when coming for their sessions. Patients who gave written informed consent filled out the CORE-OM [12] before their sessions and the Session Alliance Inventory [14] after sessions.

### Participants

The study participants were adult patients attending the psychiatric clinics. See Table 1 for a description of key socio-demographic features of our patients. We excluded participants under 18 years of age since the intention was to assess outcomes strictly for adult population.

**Table 1:** Socio-demographic characteristics

Variable	Category	Frequency (N = 345)	Percentage %
Clinic	KNH	155	48.8
	MPH	180	52.2
Therapist	Mental health workers	225	65.2
	Interns	112	32.5
	*Missing data*	8	2.3
Sex of the client	Male	249	72.2
	Female	94	27.2
	*Missing data*	2	0.6
Age of the client	Mean; SD; range	28.9; 9.9; 18–60	
Age category, years	18–27	194	56.2
	28–37	78	22.6
	> 37	73	21.2
Previous therapy	Yes	55	15.9
	No	284	82.3
	*Missing data*	6	1.7
Current psychotropic prescription	Yes	171	49.6
	No	155	44.9
	*Missing data*	19	5.5
Primary diagnosis	Addictions	188	54.8
	Psychosis	60	17.5
	Depression	58	16.9
	Anxiety/stress	41	11.9
	Interpersonal problems	22	6.4
	Physical problems	22	6.4
	Work/academic	22	6.4
	Other problems	22	6.4
	Self-esteem	17	5.0
	Trauma/abuse	16	4.7
	Personality problems	15	4.4
	Living welfare	12	3.5
	Eating disorders	11	3.2
	Bereavement/loss	08	2.3

### Therapists

We categorized our therapist sample into two distinct groups for greater clarity: mental health professionals full-time employed by the hospitals to offer mental health services and postgraduate interns. It is not uncommon to have postgraduate interns from clinical psychology, psychiatry, nursing, and counseling fields assist in delivering clinical services.

## Measures

*Clinical outcomes in routine evaluation—outcome measure* (CORE-OM; [12]. The CORE-OM is a self-report measure consisting of 34 items measuring psychological distress experienced during the preceding week, on a five-point scale ranging from 0 (“Not at all”) to 4 (“Most or all the time”). The items cover four major problem areas: wellbeing, problems, functioning, and risk (to self or others). Higher scores indicate greater distress. The CORE-OM has shown good internal and test–retest reliability (0.75–0.95), convergent validity, large differences between clinical and non-clinical samples, and good sensitivity to change [16]. A factor analysis on the present sample showed that the CORE-OM has a strong general distress factor and that the only subscale that added anything on top of that was the risk scale [16]. In the present study we used only the total score, which had excellent internal consistency (α = 0.94).

*Session Alliance Inventory* (SAI; [14]). This self-report questionnaire is based on the Working Alliance Inventory [25] and asks about the working alliance between therapist and patient in a particular session. It consists of six items that ask about therapist-patient emotional bond and agreement on goals and tasks of treatment on a Likert-type scale ranging from 0 (“Not at all”) to 5 (“Completely”). The Swedish version of the SAI has shown good reliability and validity, while the psychometric properties of the English version is still to be evaluated. We have evaluated the cross-cultural stability of the within-patient alliance effect on next-session self-reported psychological distress/symptoms [17].

### Statistical analysis

The data from this study is nested, that is, repeated measurements are nested within patients, and patients nested within therapists. For this reason, we used multilevel modeling (MLM; e.g., [49] which estimates additional variance components for each level of nesting to allow for correlation among observations belonging to the same unit (which violates the assumptions of ordinary least squares regression). Unfortunately, there was no identity information on therapists in the data (due to a mistake in data collection), so it was not possible to adjust for this level of nesting. Therefore, the model used was a two-level model with repeated observations at Level-1 and patients at Level-2. Initial explorations indicated a model including random intercept and random linear slopes of time to fit the data best. Random effects were allowed to covary and variances to differ (i.e., an ‘unstructured covariance matrix’ was estimated for the random effects). All predictors were entered as main effects and as cross-level interactions with the random slope, the latter as a way of testing their prediction of change over time.

Diagnostic information was collapsed into four larger categories: addictions, depression, anxiety/stress, and psychosis. These were the largest diagnostic groups in the sample, and the comparison group is those patients who did not fulfil any of these diagnoses. We were also interested in patient demographics (age and sex), clinic, therapist experience (mental health staff or intern). Estimation was first done using Restricted Maximum Likelihood. Due to the large number of parameters to be estimated from the relatively small dataset, we also re-estimated the models using Bayesian estimation (e.g., [21] which is more robust to issues (e.g., convergence problems and unreliable estimates) that may arise in the estimation of complex models in small samples.

## Results

Socio-demographic data along with the key clinical outcomes is summarized in Table 1. The key highlights of the results are here below.

### Age and gender of patients

The sample was predominantly male (72.2%). The mean age was 28.9 years and ranged from 18 to 60, and the majority of the participants were aged between 18 and 27 years.

### Patient attendance

The largest number of patients were from Mathare hospital (52.2%) followed by Youth clinic (40.6%), Mental Health support clinic (4.1%), and Adult Psychiatry Clinic (3.2%). The majority of the participants had no previous therapy (82.3%) and close to half (49.6%) were currently on a psychotropic medication prescription at the time of enrollment.

### Therapeutic foci

The therapeutic foci for the greatest number of patients were substance use disorders (54.8. %), psychosis (17.5%), depression (16.9%), anxiety and stress-related conditions (11.9%), interpersonal including adjustment problems (6.4%) and physical problems (6.4%), academic and work related problems (6.4%), self esteem (5%), trauma and abuse related problems (4.7%), personality problems (4.4%), eating disorders (3.2%) and other categories formed 12.2% of the cases. Mental health staff (including psychiatrists, clinical psychologists, psychiatric social workers, and mental health nurses) treated and reviewed 65.2% of our sample and the remaining 32.5% were served by student interns (psychiatry residents, postgraduate clinical psychologists, and counseling psychology students). With regards to substance use disorders we were not able to give disaggregated information about specific substances or addictions. There were several cases of poly-substance abuse that had not well documented the specific substances in the case files.

### Overall improvement in clinical outcome

The initial model without explanatory factors showed that on average CORE-OM scores decreased by 1.68 (se = 0.19, z = 8.63, *p* < 0.001, 95% CI 2.07, 1.30) every session as therapy progressed, suggesting improvement in patients’ general mental health (see Table 2).

**Table 2:** Estimates from multilevel models assessing predictors of patient change over time

	Coefficient	se	z	*p*	95% CI	
Intercept	11.78	1.10	10.68	< 0.001	9.61	13.94
Time	− 0.94	0.64	− 1.46	0.144	− 2.20	0.32
Patient support centre	2.45	2.28	1.08	0.282	− 2.01	6.91
Clinic 24	− 2.96	2.53	− 1.17	0.242	− 7.92	2.00
Mathare hospital	− 0.97	1.29	− 0.75	0.453	− 3.49	1.56
Patient support centre × time	− 0.61	1.49	− 0.41	0.681	− 3.53	2.31
Clinic 24 × time	− 1.13	1.48	− 0.77	0.444	− 4.02	1.76
Mathare hospital × time	0.23	0.70	0.32	0.746	− 1.15	1.60
Intern	*3.24*	*0.90*	*3.60*	*<* *0.001*	*1.48*	*5.00*
Intern × time	− *1.20*	*0.51*	− *2.35*	*0.019*	− *2.20*	− *0.20*
Client_Age	− 0.01	0.05	− 0.18	0.861	− 0.11	0.09
Client_Age × time	*0.08*	*0.03*	*3.02*	*0.003*	*0.03*	*0.14*
Female	*2.62*	*1.00*	*2.61*	*0.009*	*0.65*	*4.58*
Female × time	− 0.91	0.54	− 1.68	0.093	− 1.97	0.15
Previous_Therapy	1.87	1.07	1.74	0.082	− 0.23	3.97
Previous_Therapy × time	− 0.70	0.63	− 1.12	0.261	− 1.93	0.52
Current_Prescription	0.85	0.89	0.96	0.338	− 0.89	2.60
Current_Prescription × time	− 0.07	0.45	− 0.15	0.883	− 0.94	0.81
Addictions	− 0.18	1.04	− 0.18	0.859	− 2.22	1.85
Addictions × time	− 0.24	0.57	− 0.42	0.674	− 1.36	0.88
Psychosis	− 0.35	1.09	− 0.32	0.746	− 2.49	1.78
Psychosis × time	0.55	0.58	0.95	0.341	− 0.59	1.70
Depression	*2.75*	*1.11*	*2.48*	*0.013*	*0.57*	*4.93*
Depression × time	− 1.15	0.62	− 1.87	0.062	− 2.36	0.06
Anxiety	2.03	1.28	1.59	0.113	− 0.48	4.53
Anxiety × time	− 0.29	0.67	− 0.44	0.661	− 1.61	1.02

Random-effects parameters	Estimate	se	95% CI			
Time	2.22	1.00	0.92	5.37		
Intercept	37.05	7.20	25.32	54.22		
Covariance time/intercept	− 5.47	2.48	− 10.33	− 0.61		
Residual	21.99	1.66	18.97	25.49		

### Outcome prediction

We found a few significant results for the psychotherapy outcome predictions. For instance, interns in mental health units had patients who reported significantly higher initial distress (3.24 CORE-OM points, se = 0.90, z = 3.60, *p* < 0.001, 95% CI − 1.48, 5.00), and their patients reported more improvement over time (− 1.20 CORE-OM points per session, se = 0.51, z = − 2.35, *p* = 0.019, 95% CI − 2.20, − 0.20) than patients seeing mental health specialists. Older clients improved slower (0.08 CORE-OM points slower improvement per session per year for older age; se = 0.03, z = 3.02 *p* = 0.003, 95% CI 0.03, 0.14). Female patients reported higher initial distress than men (2.62 CORE-OM points, se = 1.00, z = 2.61, *p* = 0.009, 95% CI − 0.65, 4.58), and patients with depression reported higher initial distress levels (2.75 CORE-OM points, se = 1.11, z = 2.48, *p* = *0.01*, 95% CI − 0.57, 4.93) than patients with addictions, anxiety, or psychosis.

Interns had on average younger patients (Mean age for interns = 25.04, SD = 0.88, Mean age for doctors = 30.85, SD = 0.65; *t*(335) = 5.22, p < 0.001) and more patients with depression (interns saw 24.32% patients with depression as opposed to doctors who saw 13.39% patients with depression; Fisher’s exact test p = 0.02). When running the analysis on full time mental health professionals only, the result for depression disappeared but age remained a significant predictor.

### Working alliance as predictor of outcome

The working alliance measured by the SAI had a mean of 4.09 (SD = 0.83) at the first appointment. The average alliance between patient and therapist increased slightly each session up until session 3 (M = 4.33, SD = 0.70), then decreased to 4.08 (SD = 1.00) at session 6. To check the prediction of outcome by the alliance, a linear growth model (sessions nested within patients) was estimated for the CORE-OM, with the alliance at session 2 (since at session 1 the alliance might not yet be established) as level-2 predictor of intercept and linear time slope. The SAI was grand mean centered to facilitate interpretation. The results showed that at average alliance CORE-OM decreased by 1.74 points per session (se = 0.21, *p* < 0.001). For each point higher on the SAI at session 2 the CORE-OM decreased by an additional 0.58 points per session (se = 0.25, *p* = 0.02) and vice versa (i.e. with one point lower on the SAI at session 2, the CORE-OM decreased by 0.58 points less per session. The relationship between alliance and initial status on the CORE-OM was non-significant (*p* = 0.13).

## Discussion

The main finding of this exploratory work suggests that there is improvement in symptoms across the board with an 1.68 decrease in overall CORE-OM points per session attended. This suggests that by and large, the participants visiting these clinics are experiencing an improvement in their symptoms. This is an encouraging finding that patients benefit from the mental health services given the paucity of resources and specialists on ground.

In terms of associating social demographics with the treatment outcomes, we were somewhat limited in the number of factors we could tap into so as to make the study less intrusive to both the patients and the therapists.

We considered the gender and age of our participants, and in terms of therapist characteristics, we mainly considered their gender and whether they were interns (postgraduate students or residents), or fully qualified mental health professionals.

### Patient demographics

One of our key findings was greater participation of male patients than female patients. This is an unusual finding, meriting further exploration. It also differs from most data that shows men display less help-seeking behavior for psychological problems [38]. In another paper [16] validating the use of CORE-OM for Kenya, we have discussed population-specific factors, namely the pressure on the working-class Kenyans to earn their livelihood and harsh working and living conditions. It is possible that our male participants experienced significant distress that thwarted or put their livelihood functioning at risk, prompting mental health evaluation and care at the public hospitals. Additionally, this finding has also left us wondering whether women experience greater barriers and health-systems challenges [27] in seeking mental health services and if the mental health clinics are sensitive to the psychosocial and health needs of women. There is also a possibility that women present with more physical ailments that might necessitate them to visit other clinics such as Obs/Gynae, HIV/STD etc. It is likely that there is a greater footfall of women in the hospital as they are primary caregivers of elderly relatives and their own offsprings and may not have enough time or resources to attend to their own distress. Women may seek more spiritual and community resources to seek psychological relief. WHO [53] report on gender disparities in seeking mental health care notes that while women are more likely to share their problems with primary care physicians, men are much more likely to seek specialist services.

We did not collect information about socioeconomic status but know from clinical experience and existing literature that the clientele of these public hospitals largely includes people from lower socioeconomic status who cannot afford private care. The fact that the patients are, on average, improving in their mental health functioning is a helpful finding to augment existing services and integrate evidence-based interventions for the commonly seen conditions.

The most common mental health conditions in our sample include addictions (54.8%), psychosis (17.5%), depression (16.9%), and anxiety (11.9%). We noted a statistically significant difference in the distress experienced at intake and in the rate of reduction of distress by participants with depression compared to other patients. In this group we noticed the highest distress in comparison to other conditions at intake but they also improved most during treatment. This seems to be in line with research from other continents, in which depression seems to be a condition that is less chronic than many other conditions. The anxiety disorder patients, although not statistically significantly better off than the reference group, still seemed to improve fairly well during the study time. This is a group that warrants further investigation.

For patients with addictions, it may be that the CORE-OM is not the most appropriate outcome instrument, since it measures psychological distress rather than addiction per se. It is possible that patients with addictions reduce their use of alcohol and/or drugs without reducing their psychological distress. It is even possible that psychological distress increases when someone stops using drugs. When it comes to psychosis, it seems in line with clinical experience that these patients take longer time to improve than patients with less severe psychiatric problems.

Whilst we did not find differences between the two hospital samples, the settings differ. MNH has many more chronic, long-term resident patients, especially those from highly impoverished households than KNH which tends to have a mixed set of clienteles both in terms of social class and nature of mental health distress.

Another noteworthy observation is that, relatively speaking, our participants seem to be attending very few sessions; fewer than average community health clinics (mean of 5 sessions) in Western contexts [23].

### Patient progress

Females report higher distress at baseline than males, yet more males seek mental health care. This left us wondering whether these services are accessible and friendly to Kenyan women who face multifarious cultural, economic, and social challenges as discussed earlier. We will be exploring working alliance issues further to tease out whether initial encounter with a therapist might impact continuation of female patients in psychotherapy, in terms of reciprocal understanding of the distress and nature of cure. Findings on working alliance of male participants too is important and awaited. Older clients improved slower than younger ones and this is a highly neglected population in public mental health care in Kenya. Perhaps our psychotherapies need to be focusing on lifespan specific issues that might address unique needs of geriatric populations. With families increasingly moving towards nuclear units or migrating to bigger cities, the elderly population does not have specialist clinics, home or health care facility based management.

With regards to higher distress experienced in depression patients, there is a likelihood that many of these patients have had active psychosocial adversities and were managed purely on talk therapy such that the distress is considerably more at the baseline evaluation.

As analyses of intern/doctor, age and depression were all part of the same regression model, and the model controlled for the correlations among each other, we conclude that interns had better outcome than full-time mental health professionals employed by the hospitals. Poor outcomes for professionals could be due to their being overworked, lacking continuous professional education, and presence of other practical barriers.

### A silver lining

The fact that the largest cohorts were individuals seeking treatment for addictions and psychosis followed by conditions such as depression and anxiety tells us that Kenyans are seeking talk therapies for severe mental health conditions. The challenge of putting in place interventions that are culturally and time- and resource-sensitive presents itself as a compelling yet exciting opportunity for mental health professionals.

### Implications for task-sharing and mental health capacity building

Our results show that our postgraduate interns had patients with higher distress at baseline evaluation and their cases improved over time in comparison to mental health professionals working in the two hospitals. Our limited sample and fewer therapy sessions may have come in the way of making a decisive opinion here; however, it does appear that postgraduate interns might use hands-on skills in psychotherapy and psychopharmacology as they were in ongoing training. These clinics are run by interns as there is limited staff and long queues to clear. The task-sharing model of incorporating postgraduate students to augment mental health care delivery in low-resource context is compelling and could be time-and-resource efficacious. However, it can also be exploitative and not necessarily empower younger people providing these services as their efforts are not rewarded, regular supervision is not available, and interns may de facto run such high-volume clinics with little support or clinical oversight. Balancing this dynamic with a more responsible and empowerment focused task-sharing approach would create opportunities for enhanced learning for interns, provide relief and support to mental health staff, and higher number of patients may receive timely attention.

## Limitations

This study was not without its own limitations. We did not have a control group and therefore we could not ascertain whether the improvement in functioning was due to spontaneous remission. We did not have access to therapist-related demographics or covariates that might impact psychotherapy such as what therapists actually did in their sessions. We also had considerable data missing which impacted the overall outcome findings.

## Conclusions

We hope that sharing this pilot data will encourage researchers in other LMIC to study the effectiveness of psychological interventions as they are delivered in their countries. These are preliminary observations to consider for a larger sample follow-up study. Before changing practices, evaluating the existing practices by mapping clinical outcomes is a helpful route. Effectiveness-implementation studies and its hybrid designs would be important in considering the following factors when evaluating how people in low-resource contexts present mental health problems: what services are offered, from whom, at what point in treatment do mental health patients benefit from care, and how can long-term benefits be accrued through rethinking and reorganizing existing public mental health services. There is no doubt that at the end of the day with limited resources, we want time and money to be utilized effectively to develop evidence-based contextualised therapies, which can be delivered by the available staff in the minimum effective number of sessions.
